# Changes in US Veterans’ Access to Specialty Care During the COVID-19 Pandemic

**DOI:** 10.1001/jamanetworkopen.2022.32515

**Published:** 2022-09-20

**Authors:** Kevin N. Griffith, Daniel A. Asfaw, Rachel G. Childers, Andrew P. Wilper

**Affiliations:** 1 Partnered Evidence-Based Policy Resource Center, Veterans Affairs Boston Healthcare System, Boston, Massachusetts; 2 Department of Health Policy, Vanderbilt University Medical Center, Nashville, Tennessee; 3 Department of Health Law, Policy & Management, Boston University School of Public Health, Boston, Massachusetts; 4 Department of Business Administration and Economics, Presbyterian College, Clinton, South Carolina; 5 Veterans Affairs Boise Healthcare System, Boise, Idaho; 6 Department of Medicine, University of Washington, Seattle

## Abstract

This cross-sectional study identifies changes in referral volume and wait times for veterans seeking care from the specialists in the Veterans Health Administration or the community.

## Introduction

The Veterans Health Administration (VHA) began preparing for the COVID-19 pandemic in January 2020 and implemented extensive measures to reduce the spread of SARS-CoV-2 within its facilities.^1^ Measures included national screening, testing and isolation protocols, mandatory training, communication of safety information to patients, and increased telemedicine use.^1,2^ Although these measures reduced in-person visits^3^ and increased telehealth volume,^4^ their association with specialty care volume is unknown. Pandemic-related changes in referral patterns may have important implications for downstream health outcomes. In this study, we identified changes in referral volume and wait times for veterans seeking care from VHA or community-based specialists.

## Methods

This cross-sectional study was approved by the Veterans Affairs Boston Healthcare System Institutional Review Board and adhered to the STROBE reporting guideline. Informed consent was waived per institutional policy because the research could not be completed without the waiver or study alteration.

Using a previously validated algorithm,^5^ we queried the VHA Corporate Data Warehouse for all new patient referrals to specialty care from January 1, 2019, to December 31, 2021. These data include completed and cancelled referrals; exclusion of the latter may bias wait estimates downward. We calculated total appointment volume and mean (SD) wait times monthly, stratified by referral to VHA or community-based specialists. Two-sided *t* tests were used to compare changes over time, with α = .05 as our threshold for statistical significance. Analyses were conducted from October 1, 2021, through July 30, 2022, using Microsoft R Open, version 4.0.2 (Microsoft Corporation).

## Results

The VHA completed 14 516 937 internal referrals and purchased an additional 9 904 132 referrals to community-based specialists during the study period. Our sample was generally older (mean [SD] age, 63.8 [15.2] years), male (87.2%), and married (54.0%) and had some level of service-connected disability (72.2%). Our deidentified study data, which could be used to study individual specialties or subpopulations, are publicly available.^6^

**Figure 1.**
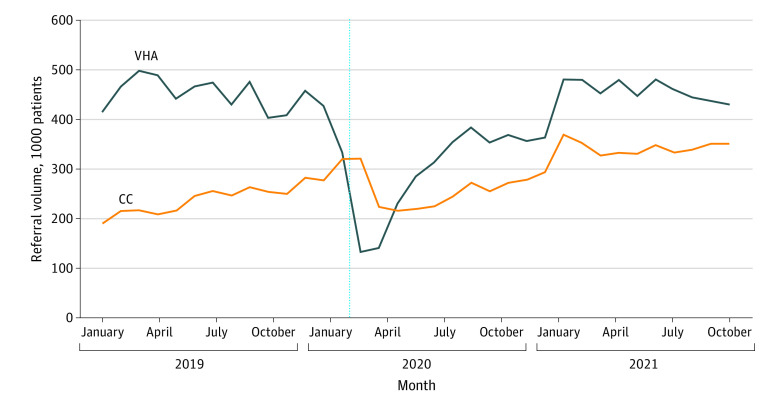
Trends in Overall Referral Volumes for Specialty Care Total specialty care referral volumes for Veterans Health Administration (VHA)–enrolled veterans are stratified by whether they were initially referred to the VHA or community care (CC). Data on all new patient referrals to specialty care were extracted from the VHA Corporate Data Warehouse. The dotted blue line indicates the declaration of a national public health emergency in February 2020.

**Figure 2.**
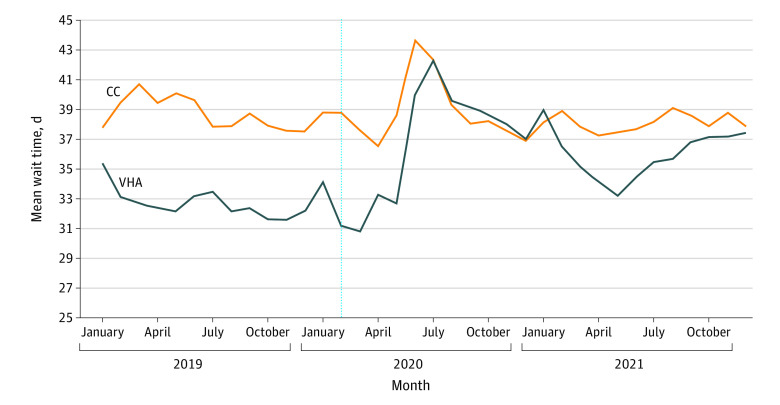
Trends in Appointment Wait Times for New Patient Referrals to Specialty Care Mean appointment wait times experienced by Veterans Health Administration (VHA)–enrolled veterans are stratified by whether they were initially referred to the VHA or community care (CC). Data on all new patient referrals to specialty care were extracted from the VHA Corporate Data Warehouse. The dotted blue line indicates the declaration of a national public health emergency in February 2020.

Before the pandemic (January 1, 2019, to January 31, 2020), VHA specialists completed a mean 452 148 (30 726) referrals each month (Figure 1 and Figure 2). Volume dropped starting in March 2020, declined by 70.7% to 132 481 referrals in April 2020, and did not fully recover until March 2021. Referrals to community-based specialists increased before COVID-19 and continued to increase until April 2020 (321 329 referrals). Community care referrals declined by 32.9% to 215 768 in June 2020, rebounding thereafter and exceeding the prepandemic baseline by March 2021.

Mean (SD) waits for community-based specialists exceeded VHA waits before the pandemic (38.6 [20.8] vs 32.8 [18.0] days; *P* < .001) and throughout the study period. Waits for VHA specialists began rising in June 2020, peaked in July 2020 (+8.2 days vs January 2020; *P* < .001) and returned to prepandemic levels by Spring 2021. Mean waits for community specialists peaked in June 2020 (+4.8 days vs January 2020; *P* < .001) and returned to their prepandemic baseline by August 2020.

## Discussion

The COVID-19 pandemic represents a unique disruption in timely access to specialty care. In this cross-sectional study, the pandemic’s onset was associated with decreased referral volume and increased waits for both VHA and community-based specialists. These changes occurred earlier and were larger among VHA specialists; this discrepancy may be attributable to the VHA’s national, early response to COVID-19, which mandated the cancellation of elective admissions, postponement of routine elective care, and implementation of social distancing measures that limited facility capacity.^1,2^ Notably, these policies were implemented even where the virus had not yet achieved community spread.

Waits and referral volume returned to prepandemic levels for VHA and community-based specialists by spring 2021. The VHA’s rapid expansion of preexisting telehealth infrastructure may explain why referral volume rebounded more quickly for VHA vs private-sector clinicians.^4^ Study limitations include the inability to identify clinical appropriateness of observed waits or mechanisms underlying the observed changes in referral volume or waits. Future work should examine the health consequences of these delays in care due to COVID-19.
